# Interest of preoperative immunonutrition in liver resection for cancer: study protocol of the PROPILS trial, a multicenter randomized controlled phase IV trial

**DOI:** 10.1186/1471-2407-14-980

**Published:** 2014-12-18

**Authors:** Oriana Ciacio, Thibault Voron, Gabriella Pittau, Maité Lewin, Eric Vibert, René Adam, Antonio Sa Cunha, Daniel Cherqui, Astrid Schielke, Olivier Soubrane, Olivier Scatton, Chady Salloum, Daniel Azoulay, Stéphane Benoist, Perrine Goyer, Jean-Christophe Vaillant, Laurent Hannoun, Emmanuel Boleslawski, Hélène Agostini, Didier Samuel, Denis Castaing

**Affiliations:** Centre Hépato-biliaire, Paul Brousse Hospital - APHP, 12-14 Avenue Paul Vaillant Couturier, 94800 Villejuif, France; Department of Radiology, Paul Brousse Hospital – APHP, 12-14 Avenue Paul Vaillant Couturier, 94800 Villejuif, France; Department of Hepato-biliairy Surgery and Liver Transplantation, Saint Antoine Hospital - APHP, 184 Rue du Faubourg Saint-Antoine, 75012 Paris, France; Department of Digestive Surgery, Mondor Hospital - APHP, 51 Avenue du Maréchal de Lattre de Tassigny, 94010 Créteil, France; Department of Digestive Surgery, Kremlin-Bicêtre Hospital - APHP, 78 rue du Général Leclerc, 94270 Le Kremlin-Bicêtre, France; Department of Digestive and Hepato-Pancreato-Biliary Surgery, Pitié-Salpetrière Hospital - APHP, 47-83 Boulevard de l’Hôpital, Paris, 75013 France; Department of Digestive Surgery and Transplantation, University Hospital of Lille, 2 Avenue Oscar Lambret, 59000 Lille, France; Clinical Research Unit Paris Sud, Bicêtre Hospital - APHP, 78 Rue du Général Leclerc, Le Kremlin Bicêtre, 94275 France; UMR-S785 Inserm, Villejuif, France; UMR-S776 Inserm, Villejuif, France

**Keywords:** Liver resection, Hepatectomy, Immunonutrition, Liver metastases, HCC, Cholangiocarcinoma

## Abstract

**Background:**

Malnutrition is an independent risk factor of postoperative morbidity and mortality and it’s observed in 20 to 50% of surgical patients. Preoperative interventions to optimize the nutritional status, reduce postoperative complications and enteral nutrition has proven to be superior to the parenteral one. Moreover, regardless of the nutritional status of the patient, surgery impairs the immunological response, thus increasing the risk of postoperative sepsis. Immunonutrition has been developed to improve the immunometabolic host response in perioperative period and it has been proven to reduce significantly postoperative infectious complications and length of hospital stay in patients undergoing elective gastrointestinal surgery for tumors. We hypothesize that a preoperative oral immunonutrition (ORAL IMPACT®) can reduce postoperative morbidity in liver resection for cancer.

**Methods/design:**

Prospective multicenter randomized placebo-controlled double-blind phase IV trial with two parallel treatment groups receiving either study product (ORAL IMPACT®) or control supplement (isocaloric isonitrogenous supplement - IMPACT CONTROL®) for 7 days before liver resection for cancer. A total of 400 patients will be enrolled. Patients will be stratified according to the type of hepatectomy, the presence of chronic liver disease and the investigator center. The main end-point is to evaluate in intention-to-treat analysis the overall 30-day morbidity. Secondary end-points are to assess the 30-day infectious and non-infectious morbidity, length of antibiotic treatment and hospital stay, modifications on total food intake, compliance to treatment, side-effects of immunonutrition, impact on liver regeneration and sarcopenia, and to perform a medico-economic analysis.

**Discussion:**

The overall morbidity rate after liver resection is 22% to 42%. Infectious post-operative complications (12% to 23%) increase the length of hospital stay and costs and are responsible for a quarter of 30-day mortality. Various methods have been advocated to decrease the rate of postoperative complications but there is no evidence to support or refute the use of any treatment and further trials are required. The effects of preoperative oral immunonutrition in non-cirrhotic patients undergoing liver resection for cancer are unknown. The present trial is designed to evaluate whether the administration of a short-term preoperative oral immunonutrition can reduce postoperative morbidity in non-cirrhotic patients undergoing liver resection for cancer.

**Trial registration:**

Clinicaltrial.gov: NCT02041871.

## Background

Hepatic resection is the treatment of choice for selected patients with benign and malignant hepatobiliary disease. Malignant tumors represent about 63 to 90% of whole liver surgery [1, 2] and the most common diagnosis is metastatic colorectal cancer [3, 4]. Primary hepatic and biliary cancers account for about 20% of liver resection, of which hepatocellular carcinoma (HCC) is the most common [1]. Only in 13 to 30% of hepatectomies an underlying liver disease, such as cirrhosis, is present. Over the past decade, many large series have documented an improvement in perioperative results, with operative mortality rates after liver resection typically less than 5% in high-volume centers. Actually liver resections are associated with about 3.5% risk of 30-days mortality [5]. The overall morbidity rate, reported in different series, is from 22 to 42% of which 10% to 15% are major post-operative complications resulting in a prolongation of hospital stay [1, 6, 7]. The most common complications are bile leak, post-operative infections, liver failure, renal failure, cardio-vascular complications and hemorrhage. About 12% to 23% of patients undergoing liver resection develop infectious complications including chest and urinary tract infections, wound infections and infected abdominal collections [6, 8]. The infectious complications account for approximately a quarter of 30-day mortality.

The negative impact of postoperative complications (POC), and specially infectious complications, on long-term outcomes after liver surgery has been widely reported for colorectal liver metastases (CLM) as well as for HCC [7, 9–12]. Various methods have been advocated to decrease the rate of infectious postoperative complications after liver resections. These include systemic interventions such as antibiotics in peri-operative period [8, 13], topical interventions such as povidone iodine gel at the time of wound closure [14] and methods to improve general health and the immunity of the individual such as prebiotics and probiotics [15, 16] and the recombinant bactericidal-permeability increasing protein in peri-operative period [17]. A recent meta-analysis of the Cochrane collaboration [18] has selected 7 randomized clinical trials from more than 1800 records identified in the literature, to determine benefits and harms of the different interventions in decreasing the infectious complications and improving the outcomes after liver resections. In any of the compared interventions there was no significant difference between the two groups in terms of mortality, numbers of serious adverse events and intensive therapy unit stay. Author’s conclusion is that till now there is no evidence to support or refute the use of any treatment and further trials are required.

To date, the effects of preoperative oral immunonutrition (ORAL IMPACT®) in non-cirrhotic patients undergoing liver resection for cancer are unknown. As seen in major gastrointestinal surgery, this treatment could significantly reduce postoperative infectious complications, length of hospital stay and care costs [19–22]. Therefore the present trial is designed to evaluate whether the administration of a short-term preoperative oral immunonutrition can reduce postoperative morbidity in non-cirrhotic patients undergoing liver resection for cancer.

## Methods/design

### Protocol overview

The PROPILS trial is a prospective multicenter randomized placebo-controlled double-blind phase IV trial with two parallel treatment groups receiving either study product (ORAL IMPACT®) or control supplement (isocaloric isonitrogenous supplement - IMPACT CONTROL®) for 7 days before liver resection for cancer. Patients will be stratified according to the type of hepatectomy (major or minor hepatectomy), the presence of a chronic liver disease and the investigator center. The main end-point is to determine in intention-to-treat analysis, the impact of immunonutrition on the overall morbidity within 30 postoperative days. Secondary end-points will be the impact of immunonutrition on postoperative 30-day infectious and non-infectious morbidity, length of antibiotic treatment, length of hospital and ICU stay, modifications on total food intake, compliance to treatment and side-effects of immunonutrition, impact on liver regeneration and sarcopenia. At last, an ancillary study will be performed to evaluate whether the supplementary costs associated to preoperative immunonutrition could be counterbalanced by its impact in care-costs.

This study is planned for a 32-month duration with a 30-month inclusion period and is registered on clinicaltrial.gov website (NCT02041871).

### Inclusion criteria

PROPILS will include adult patients undergoing planned elective liver resection for malignant tumors. The inclusion criteria are as follows: 1) liver resection for malignancies 2) including at least 1 segment resected or 3 wedge resections 3) for patients who are over 18 years of age 4) and provide a signed written consent form (Table 1).

**Table 1:** **Selection criteria of study population**

Inclusion criteria:	- Patient older than 18 years old
	- Planned elective liver resection for malignant tumour
	- At least 1 segment resected or 3 wedge resections
Exclusion criteria:	- Patient younger than 18 years old
	- Liver resection for benign lesion
	- Liver resection associated with biliary tract surgery
	- Liver resection associated with gastro-intestinal surgery
	- Cirrhosis, defined by transient elastography or by liver biopsy
	- Renal failure defined by hemodialysis
	- Pregnancy
	- History of hypersensitivity to arginine, omega-3 fatty acids, or nucleotides
	- Inability to take oral nutrition
	- Mental condition rendering the subject unable to understand the nature, end-points and consequences of the trial

### Exclusion criteria

All patients who do not meet all the inclusion criteria will be excluded. The other exclusion criteria include liver surgery associated with biliary surgery or gastro-intestinal surgery, liver cirrhosis, defined by transient elastography (Fibroscan) or by liver biopsy, renal failure defined by hemodialysis, pregnancy, history of hypersensitivity to arginine, omega-3 fatty acids, or nucleotide, inability to take oral nutrition and mental condition rendering the subject unable to understand the nature, end-points and consequences of the trial (Table 1).

### Endpoints of trial

The primary endpoint of this trial will be the rate of overall complications classified in grade II-III-IV or V according to Dindo-Clavien classification [23] in the first 30 postoperative days (POD).

The secondary endpoints include:

i) The rate of infectious complications classified in grade II, III, IV or V according to Dindo-Clavien classification in the first 30 PODs including:
  – Wound infection defined as any redness/ tenderness of surgical wound with discharge of pus
  – Abdominal abscess defined as deep collection of pus
  – Pulmonary tract infection characterized by abnormal chest X-ray with fever (>38°C) and WBC > 12.000 cells/mm^3^ and positive sputum or bronco-alveolar lavage.
  – Urinary tract infection defined as more than 10^7^ microorganisms per mL of urine
  – Bacteremia determined by two consecutive positive blood cultures without shock
  – Septic shock defined as positive blood cultures with circulatory insufficiency
ii) The length of antibiotics treatment (in days)
iii) The rate of non-infectious complications classified in grade II, III, IV or V according to Dindo-Clavien classification in the first 30 PODs including:
  – Postoperative biliary leak defined by the International Study Group of Liver Surgery (ISGLS) [24] as a bilirubin concentration in the drain fluid at least 3 times the serum bilirubin concentration on or after postoperative day 3 or as the need for radiologic or operative intervention resulting from biliary collection or bile peritonitis
  – Post-operative liver failure defined according the’ 50-50 criteria’ [25]: PT < 50% and total bilirubin >50 μmol/ml at POD 5.
  – Postoperative bleeding defined as the necessity of blood transfusion (*X*2 units) [26]
  – Respiratory failure characterized by the presence of dyspnea and respiratory rate >35/min or PaO2 < 70 mmHg
  – Circulatory insufficiency determined by unstable blood pressure requiring use of extra fluids and/or cardiac stimulants
  – Renal dysfunction defined by increase of serum urea and/or creatinine level (50% above baseline)
  – Renal failure defined as the necessity of hemodialysis
  – Multiple Organ Dysfunction Syndrome (MODS) characterized as a state of physiological derangement in which organ function is not capable of maintaining homeostasis
  – Wound dehiscence defined as any dehiscence of the fascia longer than 3 cm
iv) The length of hospital and ICU stay (in days)
v) Post-operative liver regeneration: all patients will undergo 4 successive volumetric helical computed tomography estimations of their liver volumes before surgery (day 0) then at POD2 (day 16), POD10 (day 24) and POD30 (day 44). Preoperative measurement of the future remnant liver will be performed using as landmarks hepatic vascular structures, identified by bolus injection of contrast, and the gallbladder. Post-operative measurements will be performed for the whole remnant liver and could be realized without injection of contrast. A volumetric assessment at POD 2 has been considered necessary to clearly estimate the volume of remnant liver. Liver regeneration at POD 10 (LR10) and 30 (LR30) are calculated by using the following formula, after assuming that the density of liver was close to 1:
vi) Sarcopenia: all patients will undergo 2 helical computed tomography estimations of psoas muscle area at the level of L3-L4 before surgery (day 0) then at POD30 (day 44).
vii) Estimation of modifications on total food intake, compliance to immunonutrition treatment and side effects of immunonutrition. During treatment period, patients will be asked to fill in a formulary with an evaluation of food intake per day.

### Treatments administered

After inclusion, patients will be randomized in two arms:

Arm A: immunonutrition (ORAL IMPACT®)

Arm B: isocaloric isoprotidic nutritional support (IMPACT CONTROL) that has the same composition of ORAL IMPACT, but does not contain the immunonutriments (RNA, omega-3 fatty acids and arginine).

Oral immunonutrition (ORAL IMPACT®) and isocaloric isonitrogenous control supplement (IMPACT CONTROL) will be produced by NESTLE Nutrition, France.

In both groups, patients will be asked to drink three 74 g sachets of the product daily for 7 days before surgery. Both study product (ORAL IMPACT®) and control product (IMPACT CONTROL®) will be presented in the same form and appearance (powder) including the packaging material. The table below reports the composition of each product (Table 2).

**Table 2:** **Composition of Oral Impact**® **and Impact Control**® **supplement (in powder form)**

Content (1 sachet)		Oral Impact® (74 g)	Impact Control® (74 g)
**Energy content**	kCal	303	303
	kJ	1275	1275
**Proteins**	g	16.8	16.8
- L-Arginine	g	3.8	0
- L-Arginine + Glutamine	g	2.46	0
**ARN**	g	0.45	0
**Nitrogen**	g	3.3	3.3
**Glucids**	g	40.2	40.2
**Lipids**	g	8.3	8.3
- Omega-3 fatty acids	g	1	0
- EPA-DHA	g	1	0
Fibres	g	3	3
Sodium	mg	320	320
Potassium	mg	402	402
Calcium	mg	240	240
Magnesium	mg	69	69
Phosphorus	mg	216	216
Chloride	mg	360	360
Iron	mg	3.6	3.6
Zinc	mg	4.5	4.5
Copper	mg	0.5	0.5
Manganese	mg	0.6	0.6
Selenium	ug	14	14
Fluoride	mg	0.51	0.51
Iodine	ug	45	45
Vitamin A	ug	300	300
Vitamin D3	ug	2	2
Vitamin E	mg	9	9
Vitamin K	ug	20	20
Vitamin B1	mg	0.36	0.36
Vitamine B6	mg	0.45	0.45
Vitamin B9	ug	60	60
Vitamin B12	ug	1.7	1.7
Vitamin C	mg	65	65
**Osmolarity**	mOsm/L	477	477

### Data collection and follow up

Patients will be followed-up for 44 days (POD30) and all data collected by investigating physician will be entered in a computerized case report forms. These recorded data are summarized in Table 3.

**Table 3:** **Data collected during the study**

	1st consultation	D0	D7 to D12	D13	D14	D15	D16	D17	D19	D21	D24	D44
						POD1	POD2	POD3	POD5	POD7	POD10	POD30
**General data**												
First Name	x											
Last Name	x											
Sexe	x											
Date of birthday	x											
Phone number	x											
Adress	x											
Center	x											
Medical history and comorbidity	x											
Concomittant medication	x											
Preoperative chemotherapy	x											
History of liver surgery	x											
ASA score		x										
Date of 1st consultation	x											
Operating date					x							
Type of liver resection					x							
**Preoperative protein-energy malnutrition**												
Height	x											
Actual weight	x			x							x	x
BMI	x			x							x	x
Usual weight	x											
% of loss of weight in one and 6 months	x											
Albumin, prealbumin		x		x								
MNA-SF (for patients > 70 years old)	x											
Sarcopenia (Psoas area on CT scan)		x					x				x	x
**Compliance to immunonutrition treatment**												
Nutritional journal				x								
**Liver function assessment**												
PT, INR		x		x		x		x	x	x	x	x
AST, ALT		x		x		x		x	x	x	x	x
GGT, PAL		x		x		x		x	x	x	x	x
Total and direct bilirubin		x		x		x		x	x	x	x	x
Platelets		x		x		x		x	x	x	x	x
Volumetric assessment of (future) remnant liver	x						x				x	x
**Renal function assessment**												
Creatinine, urea		x		x		x		x	x	x	x	x
Sodium, potassium		x		x		x		x	x	x	x	x
Hemoglobin		x		x		x		x	x	x	x	x
White cells, lymphocytes		x		x		x		x	x	x	x	x
**Follow up**												
Overall morbidity												x
Infectious complications												x
Biliary leak												x
Length of hospital and ICU stay												x
Length of antibiotic treatment												x

Preoperative data including age, sex, medical history and comorbidities, concomitant medication, preoperative chemotherapy, history of liver surgery will be collected during the first consultation, for inclusion in the study. In addition, an evaluation of the nutritional status of the patient will be realized by collecting the following data: weight and BMI, albumin and prealbumin values, evaluation of sarcopenia on CT Scan and, for patients older than 70 years old, the MNA-SF test.

After randomization patients will receive either preoperative immunonutrition by Oral Impact® or preoperative nutritional support without immunonutriments (Impact control®) for 7 days before liver surgery. To evaluate the compliance to preoperative immunonutrition, its side effects and its impact on total food intake, patients will be asked to fill out a nutritional journal during this period.

Liver surgery will be performed by laparotomy or laparoscopy according to the decision of the surgeon.

During postoperative period, monitoring of patients will no differ from conventional monitoring after liver surgery, including daily physical examination, blood analyses at postoperative day (POD) 1, POD3, POD5, POD7 and POD10, abdominal CT scan at POD10. In addition abdominal CT scan will be done at POD2 to accurately assess the liver volume immediately after hepatectomy. Monitoring and management of postoperative complications are left to the discretion of the clinicians in charge of the patient. All complications will be collected as soon as possible, during hospitalization, and classified according to the classification of Dindo and Clavien [23].

**Figure 1:**
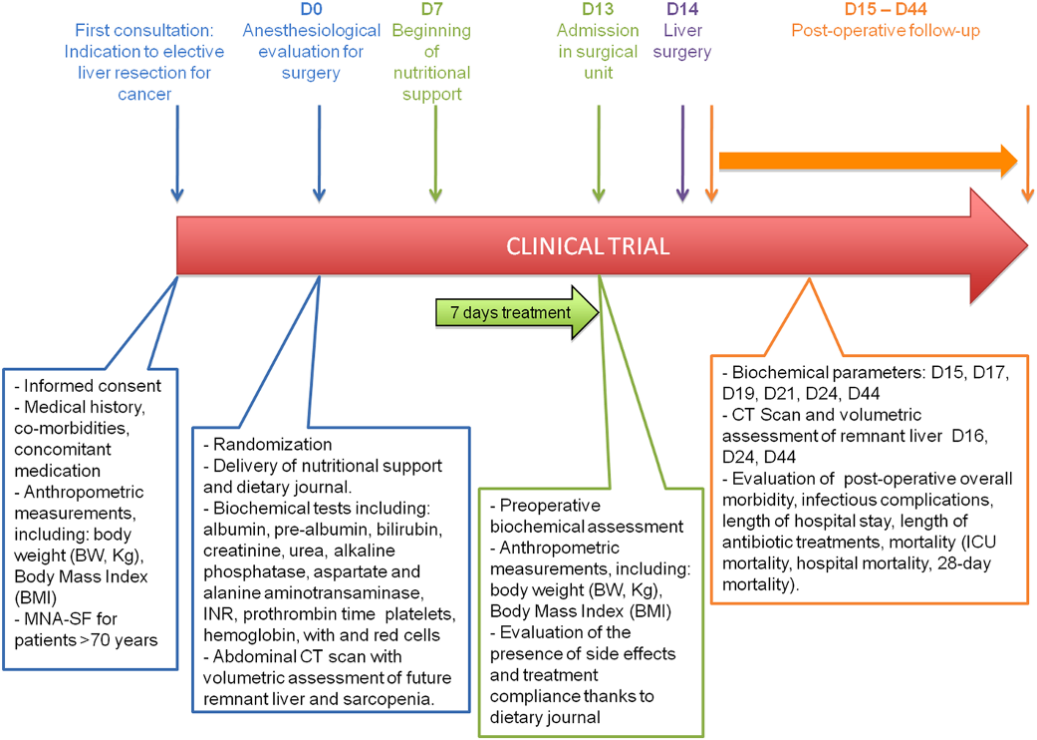
**Schema depicting the workflow of the study.**

Patients will be systematically reviewed at day 44 with abdominal CT scan to identify postoperative complications after discharge, evaluate liver regeneration and sarcopenia and to terminate their participation to the study (Figure 1).

Concerning the radiological investigations performed preoperatively and then on POD2, POD10 and POD30, the assessment of remnant liver volumes and of sarcopenia, will be performed by a single radiologist.

### Randomization

Patients will be randomized in blocks, with a distribution of 1:1 for the control group and experimental group. The block size will be random and will be informed in the report of the study. Randomization will be stratified by investigator centre, type of hepatectomy and presence of an underlying hepatopathy.

The randomization list will be established using the software NQuery Advisor®v6.01, a validated system using a generator of pseudo-random numbers, so that the sequence of treatments is both repeatable and non-predictable. The physician-investigator will enter data for inclusion in computerized case report forms (eCRF), implemented using the software “Cleanweb” (Telemedicine technologies). The physician-investigator will be able then to access the randomization module of the software that will award the group in which the patient is randomized. A unique identification alphanumeric number will be assigned to each patient: “number of the center (3 characters) – Number of inclusion in the center (3 characters) - Initials of first and family names (1 and 1 characters) - randomization group”. Patients who left the study keep their number included if it has already been given. New patients will always receive a new issue of inclusion.

The randomization list will not be known in advance by the investigators. The statistical analysis and preparation of tables and graphs for the report of the study by the statistician of the study will be blinded to the extent possible. The unblinding may take place only after all data has been entered into the database of the study, all requests have been closed and the database has been frozen by the Data Manager of the study.

If necessary, unblinding may be performed according to validated procedures of the promoter. Access to randomization codes during the phase of blinding will be monitored and documented and the documentation will be kept in the “CTMS (Clinical Trial Management System)”.

### Participating centers

Six French centers will participate in the study: the Paul Brousse University Hospital in Villejuif, the Saint Antoine University Hospital in Paris, the Mondor University Hospital in Creteil, the Kremlin-Bicêtre University Hospital, the Pitié-Salpetrière Hospital in Paris, the University Hospital in Lille.

### Statistical methods

#### Sample size calculation

The hypothesis of this phase IV trial is that immunonutrition will reduce overall postoperative 30-day morbidity rate. The sample size calculation is based on the detection of significant difference in the primary end-point parameter of the trial. We assumed a postoperative complication rate of 36% in the conventional group (Arm B) according with several studies about complication rate after liver surgery. A reduction of 33.3% would be considered to indicate the efficacy of treatment. With an expected complication rate of 24% in the immunonutrition group (Arm A), the sample size necessary for the trial with a power of 80% and a one-sided significance level of 0.05 was calculated to be 180 patients per group. An assumed 10% drop-out rate in this trial (due to non-compliance, intolerance, etc.) will raise the sample size to 198 patients per group. Therefore, at least a total of 400 patients (200×2) have to be included to the trial.

#### Statistical analyses

The statistical analysis will be based on the intention-to-treat principle with one-sided test for the primary and secondary endpoints. However, attempts will be made to analyse “per protocol”, “completer”, and “intent-to-treat” populations separately, when statistically appropriate. A p < 0.05 will be considered as significant.

First of all, an establishment of patient flow chart according with CONSORT 2010 statement [27] will be realized. This one will describe precisely the progress through the different phases of the randomized trial (enrolment, intervention, allocation, follow-up and data analysis).

Secondary, a description of demographic and clinic features of patients in the 2 groups will be calculated by using valid number, frequency count and percentage for categorical data and by using mean, standard deviation, 95%-confidence interval of the mean, minimum lower quartile, median and upper quartile for continuous data.

To study primary end-point (that is the rate of postoperative complication grading II, III, IV or V in Dindo-Clavien’s classification), Pearson Chi-square test or Fisher’s exact test will be used when appropriate.

To study secondary end-points, the Pearson Chi-square or Fisher’s exact test will be used to compare categorical variables between the 2 groups, and the independent-samples *t*-test will be used to compare continuous variables. A multivariate analysis will complete this statistical plan.

### Ethical matters

This study is conducted according to the principles of the declaration of Helsinki and the principles of the Good Clinical Practices guidelines. This study was approved by ethics committee ‘Ile de France 1 (IDF1)’ of the Hotel-Dieu Hospital on May 2013 under the registration number 2013-A00481-44. Approval from the ‘Ile de France 1 (IDF1)’ ethics committee of the Hotel-Dieu Hospital is sufficient for the 6 study centers (Paul Brousse University Hospital, Saint Antoine University Hospital, Mondor University Hospital, Pitié-Salpetrière University Hospital, Kremlin-Bicêtre Hospital and the University Hospital in Lille).

The study has also been approved by the ANSM (Agence nationale de sécurité du medicament et des produits de santé) on May 2013.

This trial has been registered on Clinicaltrial.gov website under the identification number NCT02041871.

The institutional promoter is the Paul Brousse University Hospital, Villejuif, France. This study received a grant from the French National Cancer Institute (Institut National Cancer - INCa) in 2012 and the study protocol has undergone peer-review by the funding body.

The study products (ORAL IMPACT and IMPACT CONTROL) were donated by NESTLE Clinical Nutrition, France.

Informed consent will be obtained from each patient in a written form before enrolment and randomization.

### Study status

This study is currently collecting data and there has not been any publication concerning the analysis of the data collected until today.

## Discussion

The nutritional management is a key element to consider in surgical patients. Protein-energy malnutrition (PEM) is an imbalance between the intake of nutrients by an organism and the needs and expenditure of these. The prevalence of PEM in general surgery and oncological units is high (20% up to 50%) [28–30]: malnutrition was found in 17% to 46% of patients in general surgery [31–34], in 55% to 80% of patients with gastrointestinal cancers and up to 70% of the patients in the waiting list for liver transplantation [35]. Despite the high prevalence of PEM in hospital patients and above all in general surgery and oncological units, malnutrition remains unappreciated and neglected by clinicians and can be further aggravated by hospitalization, treatments and surgical procedures [36].

Several studies have shown that PEM significantly impairs postoperative course and increases morbidity [37–39], in particular infectious complications, mortality [40, 41], length of stay and costs [42, 43] after surgical procedures. Moreover surgical stress, which is an acute injury, increases metabolic needs and results in release of cytokines, which worsen anorexia and muscle wasting. So malnutrition leads to increased infectious post-operative complications and surgical stress worsens malnutrition.

In malnourished patients, many studies [44, 45] have shown a benefit of nutritional support before surgery. Compared with total parenteral nutrition (TPN), enteral nutrition (EN) in patients undergoing surgery results in a significantly shorter length of hospital stay, lower incidence of any complications and infectious complications and lower sepsis scores, but no difference in mortality, as shown in the meta-analyses by Elia and colleagues [45]. A systematic screening should be instituted to identify malnourished patients and propose an appropriate and efficient nutritional support in order to reduce postoperative complications.

The immunonutrition is the use of nutrients to improve nutritional status and to modulate the immune and inflammatory responses to a stress. The concept of immunonutrition arises from the observation that surgical stress predisposes patients to immune dysfunction and from the findings that chronic disease-related malnutrition is tightly linked to the effect of an inflammatory state on metabolism. Arginine, glutamine, omega-3 fatty acids and RNA are the key nutrients and immunonutrition could be administered as enteral or parenteral nutritional supplement. Arginine plays an important role in connective tissue repair and cells proliferation. It is the precursor of nitric oxide, an important signaling molecule with cytostatic and cytotoxic effects [46]. Arginine is also an essential metabolic substrate for immune cells, involved in normal lymphocyte function, T lymphocytes multiplication and maturation [47] and in the immune response again stress and tumors [48]. Furthermore, recent preclinical study has shown that arginine supplementation could afford some protection from necrosis and apoptosis in ischemia/reperfusion liver injury [49] thus helping liver regeneration after hepatic resection. Omega-3 fatty acids are anti-inflammatory agents, which decrease the production of adhesion molecules and inflammatory mediators such as cytokines. They could reduce the intensity of the inflammatory response and modulate immune response to stress [50]. Nucleotide supplementation has been shown to improve some aspects of tissue recovery from liver ischemia-reperfusion injury or radical resection [51] and to modulate TH1/TH2 balance [52].

Several studies [19, 21, 22] have just analyzed the impact of the immunonutrition in the modulation of inflammatory response and immune function after surgical procedures. ORAL IMPACT® (Nestlé Nutrition) is the most frequently used product in these trials. In these studies immunonutrition showed a significantly decrease of postoperative infectious complications, length of hospital stay and care costs, regardless of the baseline nutritional status of the patients. The meta-analysis by Cerantola et al. [53] has selected 21 randomized controlled trials, enrolling a total of 2730 patients, from more than six-hundred records identified in the literature, to determine the impact of perioperative immunonutrition in gastrointestinal surgery: immunonutrition significantly reduced overall complications and postoperative infection when used before surgery, both before and after operation, or after surgery, led to a shorter hospital stay but had no influence on mortality. Finally in the study of Bozzetti et al. [54] on 1410 subjects undergoing major abdominal surgery for gastrointestinal cancer, nutritional support reduced morbidity versus standard intravenous fluids with an increasing protective effect of total parenteral nutrition, enteral nutrition, and immune-enhancing enteral nutrition. This effect remained valid regardless the severity of risk factors identified at the multivariate analysis and it was more evident by considering infectious complications only.

The use of immunonutrition in liver surgery has been poorly studied and till now no recommendation is available. Only one trial from Mikagi et al. [55] has evaluated the effects of immunonutrition before hepatectomy on postoperative outcomes. In this randomized controlled trial 26 patients undergoing liver resections for liver tumours were randomized to immunonutrition and control groups each consisting of 13 patients. The study failed to show any significant difference in postoperative complications or duration of postoperative hospital stay because a lack of power. Two more studies, by Fan [56] and Okabayashi [57], have analyzed the interest of a preoperative enriched nutritional support (branched chain amino acids-enriched nutrient support) for patients undergoing liver resection for hepatocellular carcinoma with cirrhosis and have shown a significant reduction of postoperative infectious complications.

To date, the effects of preoperative oral immunonutrition (ORAL IMPACT) in non-cirrhotic patients undergoing liver resection for cancer are unknown. As seen in major gastrointestinal surgery, this treatment could significantly reduce postoperative infectious complications, length of hospital stay and care costs. Therefore the present trial is designed to evaluate whether the administration of a short-term preoperative oral immunonutrition can reduce postoperative morbidity in non-cirrhotic patients undergoing liver resection for cancer.

## Abbreviations

ASA score: American Society of Anesthesiologists Score
ICU: Intensive care unit
CLM: Colorectal liver metastases
HCC: Hepatocellular carcinoma
EN: Enteral nutrition
TPN: Total parenteral nutrition
MODS: Multiple organ dysfunction syndrom
PEM: Protein-energy malnutrition
POD: Postoperative day
POC: Postoperative complications
LR: Liver regeneration
MNA-SF: Mini nutritional assessment short-form.
